# Association of plasma MMP-2 levels and prognosis of patients with intracerebral hemorrhage: a prospective cohort study

**DOI:** 10.3389/fneur.2023.1259339

**Published:** 2023-11-27

**Authors:** Wenmin Yu, Jin Peng, Zhiying Chen, Huimin Li, Jianyuan Yang, Yun Wu, Manqing Zhang, Moxin Wu

**Affiliations:** ^1^The School of Basic Medical Science, Jiujiang University, Jiujiang, Jiangxi, China; ^2^Key Laboratory of System Bio-medicine of Jiangxi Province, Jiujiang University, Jiujiang, China; ^3^Clinic College/Hospital, Jiujiang University, Jiujiang, Jiangxi, China; ^4^Department of Neurology, Affiliated Hospital of Jiujiang University, Jiujiang, China; ^5^College of Pharmaceutical and Life Sciences, Jiujiang University, Jiujiang, Jiangxi, China; ^6^Faculty of Medicine and Dentistry, University of Alberta, Edmonton, AB, Canada; ^7^Medical College of Jiujiang University, Jiujiang, China; ^8^Department of Medical Laboratory, Affiliated Hospital of Jiujiang University, Jiujiang, China

**Keywords:** matrix metalloproteinases-2 (MMP-2), intracerebral hemorrhage (ICH), edema, prognosis, inflammatory

## Abstract

**Objective:**

The role of MMP-2 in patients with ICH is controversial and the impact of plasma MMP-2 level on clinical outcome is still unclear.

**Materials and methods:**

In this study, the peripheral venous blood was acquired from 93 patients with ICH and 88 healthy controls within 24 h of hospitalization and at enrollment. We retrospectively investigated plasma MMP-2 levels of patients and healthy controls. The edema volume, the NIHSS score, the GCS score, and mRS were used to assess and quantify neurological deficit following ICH. Logistic regression analysis was configured to determine the independent relation of plasma MMP-2 levels with clinical outcomes. In addition, the plasma MMP-14 levels and complement C4 level were tested to explore the relationship with plasma MMP-2 level.

**Results:**

There was a significant reduction of plasma MMP-2 levels in ICH patients than that in healthy controls (38.02 ± 1.71 vs. 54.03 ± 2.15, *p* < 0.0001), and MMP-2 is negatively correlated with the edema volume (*r* = −0.2187, *p* < 0.05), NIHSS score (*r* = −0.2194, *p* < 0.05), blood leucocyte count (*r* = −0.2549, *p* = 0.012), and complement C4 level (*r* = −0.2723, *p* = 0.005). There is positive correlation between MMP-2 level and GCS score (*r* = 0.2451, *p* = 0.01) and MMP-14 level (*r* = 0.7013, *p* = 0.005). The multivariate analysis revealed that reduced plasma MMP-2 level is associated with elevated edema volume (OR = 0.2604, 95% CI [0.07 to 0.84], *p* = 0.02).

**Conclusion:**

The plasma MMP-2 level in patients with ICH is significantly lower than that of healthy controls, and plasma MMP-2 level may be a prognostic factor. Plasma MMP-2 levels are correlated with the clinical outcomes of patients and negatively correlated with blood leucocyte count and complement C4 level in patients with ICH.

## Introduction

Intracerebral hemorrhage (ICH) is among the most frequent cerebrovascular diseases (1). Compared with other types of stroke, it has higher morbidity and mortality; however, the treatment remains ineffective (2). The main cause of primary brain injury is the oppression and destruction of the adjacent tissues by hematoma formation (3). The literature indicates that peri-hematoma edema (PHE) during ICH can cause poor prognosis in some patients (4, 5).

Extensive studies have indicated that matrix metalloproteinases (MMPs) are essentially involved in secondary brain injury (SBI) via neuroinflammation because of disrupted blood–brain barrier (BBB), cell apoptosis, and cytotoxic and vascular brain edema after ICH (6). MMP-3, 9, and 12 have been particularly observed to be associated with ICH pathophysiology (7). MMPs disrupt the BBB, and leukocyte migrates into the brain, contributing to a peri-hemorrhagic inflammatory response (8). However, the activity and involvement of metalloproteinases-2 (MMP-2) inpatients with ICH is controversial. The early BBB opening at 3 h is associated with enhanced MMP-2, whereas late opening at 48 h is linked with elevated MMP-9 (9). MMP-2 and MMP-9 are critical for tight junction proteins degradation and BBB disruption (10). However, research conducted on MMP-2-deficient mice revealed that these mice had an alleviated rate of hemorrhagic transformation, improved neurologic activity, and smaller hemorrhage volume than the wild type (11). In terms of clinical research, there is also contradiction. One research showed that level of MMP-2 significantly decreased within 24 h in the onset of spontaneous ICH, then the level of MMP-2 begin to rise, until 3 months later, the level just returned to the level before ICH occurred (2). Another study reported that serum level of MMP-2 was decreasing after spontaneous ICH onset, but it continues to decrease until 7 days later (12). The role and mechanism of MMP-2 in ICH remains undetermined. Power showed that transcription of MMP-2 enhanced in a collagenase-mediated ICH rat model (13). Furthermore, another member of the MMP family, MMP-14 (also MT1-MMP), MMP-2, TIMP-2, and MMP-14, the three molecules come together activates the MMP-2, MMP-14 is bound to the membrane. The activity of the MMP-2 is constrained to the region close to the activation site (14).

These different results suggest that further study is needed on the role of MMP-2 in patients with ICH. This investigation elucidated the plasma MMP-2 levels in individuals who suffered ICH, its influence on clinical outcomes, and its correlations with other parameters.

## Materials and methods

### Patient characteristics

This retrospective cohort investigation included 93 patients (males = 62, females = 31, mean age ± SD = 64 ± 14 yrs.) who were diagnosed with acute spontaneous ICH by head computed tomography (CT) scans within 24 h of onset at the Affiliated Hospital of Jiujiang University (China) between January 2021 to April 2023. All the participants were hospitalized within 24 h after the stroke and received non-operative treatment for their hematomas. Individuals who (1) were < 20 years old, (2) needed surgical treatment, (3) indicated infratentorial bleeding; hematomas related to tumors, trauma, coagulation disorders, aneurysms, and vascular malformations; hemorrhagic transformation of cerebral infarction, (4) required surgical removal of hematoma after the first non-enhanced CT scan, (5) were pregnant, (6) had pre-ICH as evident by Modified Rankin Scale (mRS) points ≥ 2 before ICH, and (7) other conditions, including severe infections in the past month, autoimmune diseases, and known malignant tumors were excluded from this investigation. Age and gender matched healthy volunteers without any history of surgery, hypertension, diabetes, malignant tumor, stroke, and other diseases were selected as the control cohort, and they received laboratory examination. This investigation was authorized by the Review Boards at the Affiliated Hospital of Jiujiang University (Grant No. IRB2022-JJU-032-21). Patients or their immediate family members signed the written informed consent, whereas controls signed by themselves.

### Data collection

The peripheral venous blood was acquired from all the ICH individuals and healthy controls within 24 h of hospitalization and at enrollment, respectively. Relevant data, including gender, vascular risk factors (diabetes mellitus and hypertension), age, alcohol consumption, leucocyte count, cigarette smoking, and blood glucose and potassium levels, were recorded. To assess the volume of edema, Siemens Leonardo V software for semiautomatic CT volumetry was utilized. Hematoma location and edema volumes were elucidated, and the severity of stroke was measured via Glasgow Coma Scale (GCS) and mRs at admission to evaluate the neurological function recovery of patients after ICH.

### Laboratory procedures

The blood specimen was drawn via antecubital vein puncture under sterile conditions at the time of National Institutes of Health Stroke Scale (NIHSS) calculation 24 h after symptomatic ICH. The sample was centrifuged at ambient temperature to acquire plasma and aliquot and then kept at −70°C until subsequent analysis. Plasma MMP-2 levels were elucidated via Enzyme Linked Immunosorbent Assay (ELISA) kit (No. KE00077, Proteintech Group, Wuhan, China). According to the manufacturer’s instructions, the lower MMP-2 quantification limit was 0.05 ng/mL. ELISA Kit for Matrix Metalloproteinase 14 (MMP14) (No. SEC056Hu, Cloud-Clone Corp, Wuhan, China), according to the manufacturer’s instructions. According to the manufacturer’s instructions, the lower MMP-14 quantification limit was 1.56 ng/mL. Level of plasma complement C4 was determined by turbidimetric measurements using complement C4 assay kit (No. 20230712, Kehua Bio-engineering Co., Ltd., Shanghai, China) (15). According to the manufacturer’s instructions, the lower C4 quantification limit was 0.16 g/L.

### Statistical measurements

All the statistical measurements were carried out on GraphPad Prism v9.0 (GraphPad Software Inc., La Jolla, CA, United States). Kolmogorov–Smirnov test was conducted to determine normal distribution of quantitative data. The intergroup substantial differences were assessed via the Fisher exact test for categorical variables, the χ2 test for qualitative data and the Mann–Whitney U test for two groups comparison. The Kruskal-Wallis H test was carried out for multi-group comparison. The categorical variables are expressed by the number of cases (percentage). The GCS and NIHSS scores and the median hematoma volume were defined as cutoff values, respectively. Bivariate correlation was analyzed via the Spearman coefficient, and the correlation of plasma MMP-2 concentration with clinical results was elucidated by multivariate logistic regression. *p* < 0.05 was termed statistically essential.

## Results

### Patient selection and subject characteristics

**Figure 1:**
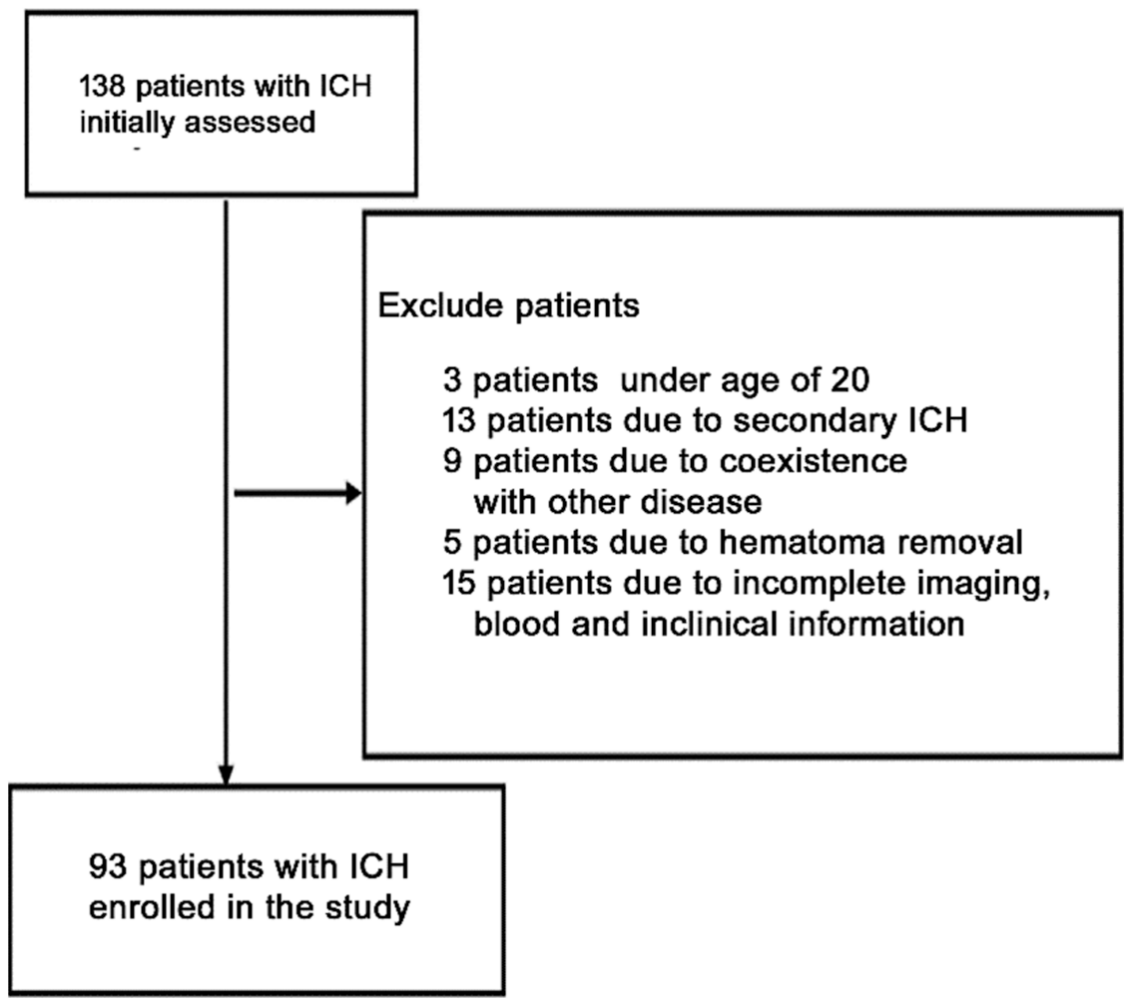
Flowchart for screening eligible patients with acute spontaneous ICH. Initially, 168 patients with ICH were evaluated. Since then, 75 patients were excluded. Finally, 93 patients with ICH were enrolled.

Initially, this investigation included 138 acute spontaneous ICH individuals who were hospitalized within 24 h after symptoms onset. However, based on the exclusion parameters, 65 patients were removed from the study, leaving 93 ICH individuals (males = 60 and females = 33) (Figure 1). Additionally, 88 healthy controls (males = 48 and females = 40) were recruited. The median ages of ICH and healthy participants were 64 ± 14 and 64 ± 9 yrs., respectively. No statistically substantial difference associated with gender (*p* = 0.249), current smokers (*p* = 0.74), age (*p* = 0.724), and alcohol drinkers (*p* = 0.99) was observed between ICH and healthy cohorts. Furthermore, as expected, the ICH cohort had an elevated proportion of hypertensive and diabetic (*p* < 0.001) individuals with substantially increased plasma glucose concentration (9.20 ± 3.05 vs. 6.36 ± 1.99, *p* < 0.001) and blood leucocyte count (9.50 ± 4.29 vs. 6.36 ± 1.62, *p* < 0.001) than a healthy cohort. Table 1 depicts the patients’ demographic and clinical characteristics.

**Table 1:** Demographic data and vascular risk factors of the patients with ICH and control in this study.

	ICH(93)	Control(88)	*p*
Male (*n*)	60	48	0.249
Age, years (mean ± SD)	63 ± 14	64 ± 9	0.7248
Hypertension (*n*)	67	0	<0.001**
Diabetes mellitus (*n*)	13	0	<0.001**
Current smoking (*n*)	4	5	0.74
Alcohol consumption (*n*)	14	13	0.99
Plasma glucose level (mmol/L)	9.2 ± 3.05	6.26 ± 1.99	<0.001**
Blood leucocyte count (×10^9^/L)	9.50 ± 4.29	6.36 ± 1.62	<0.001**

The *n* refers to the number of patients in group, respectively. Age was reported as the mean ± standard deviation. Intergroup comparisons of various variables were conducted using the χ2 test for qualitative data, and Mann–Whitney U-test for quantitative data. The asterisk indicates statistical significance (*p < 0.05, **p < 0.01).

### Reduced plasma MMP-2 levels and their correlation with clinical outcomes

**Figure 2:**
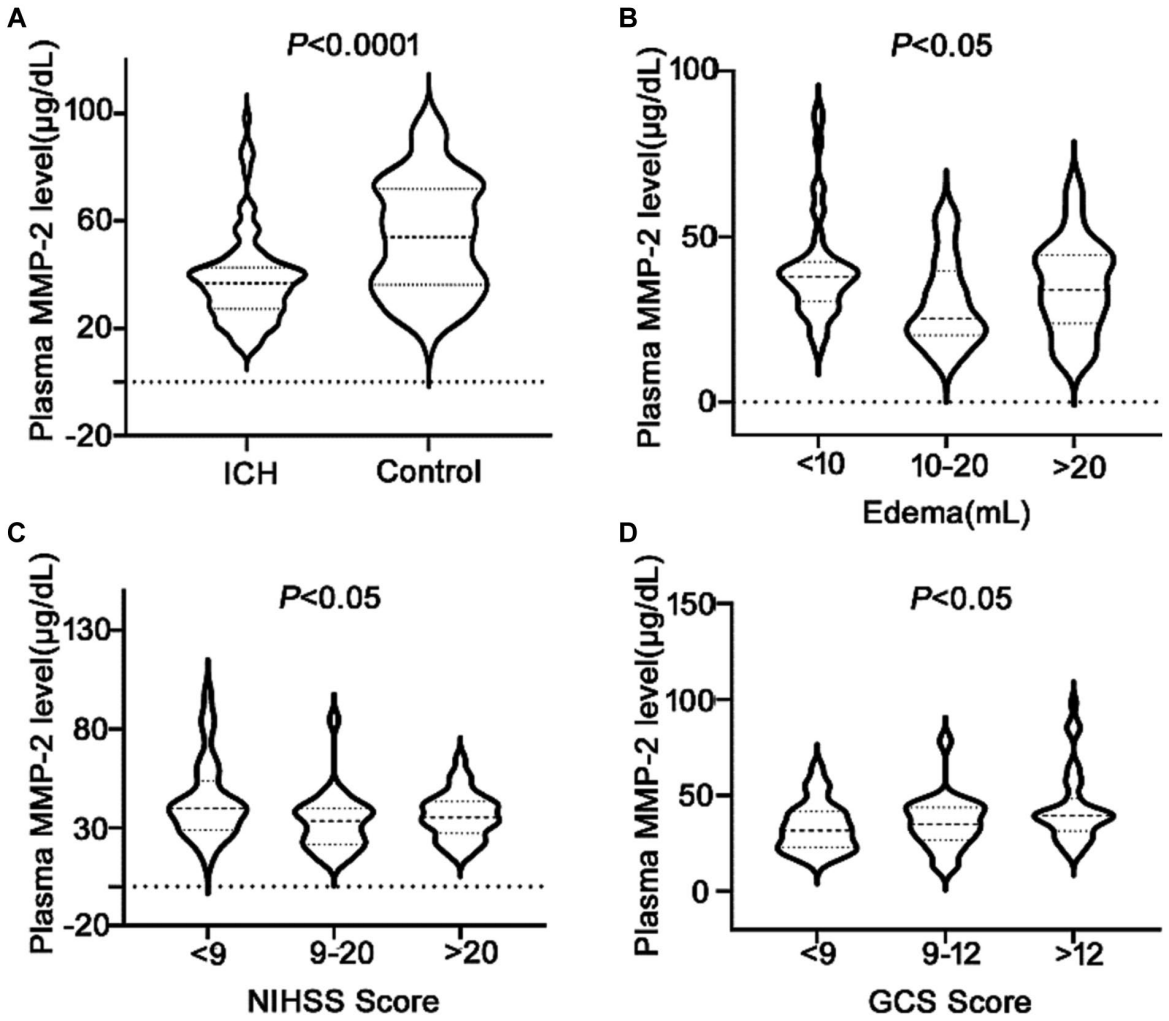
Low plasma MMP-2 levels in ICH patients and differences of MMP-2 levels between groups. **(A)** Plasma MMP-2 level in ICH and healthy controls. The differences of plasma MMP-2 levels between groups in **(B)** edema volume, **(C)** NIHSS score, and **(D)** GCS score after acute ICH patients. The Mann–Whitney U test was carried out for two groups comparison in **(A)**. The Kruskal-Wallis H test was carried out for multigroup comparison in **(B–D)**. The asterisk indicates statistical significance (*p < 0.05, **p < 0.01).

**Figure 3:**
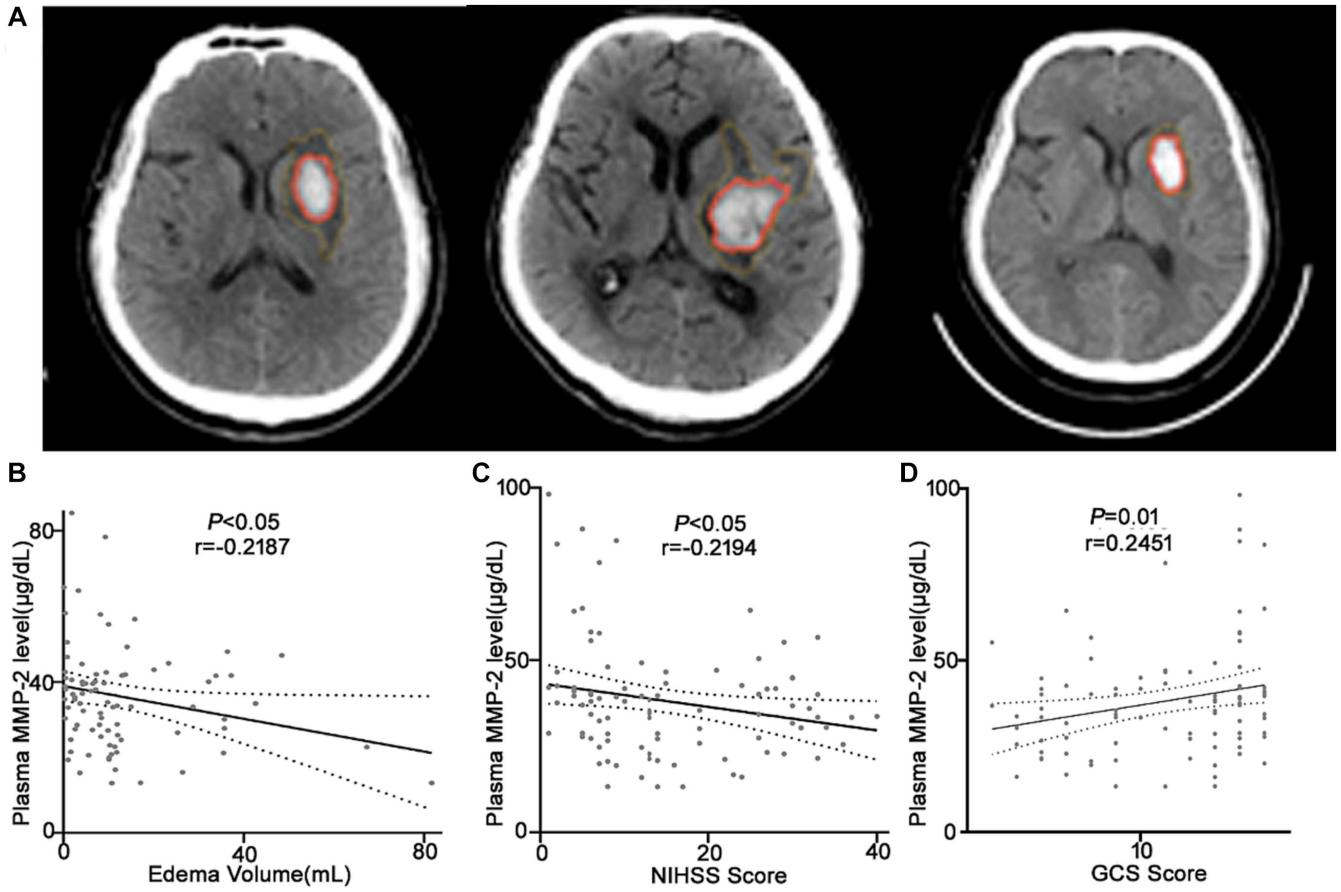
Plasma MMP-2 levels are negatively correlated with the clinical outcomes of the ICH patients. **(A)** CT images of the ICH patients used for analysis. Correlation analysis between the plasma MMP-2 level and **(B)** edema volume, **(C)** NIHSS score, and **(D)** GCS score of the ICH patients (*n* = 93). Correlations were done using the Spearman’s correlation coefficient in ICH, plasma MMP-2 levels were closely correlated with edema volume, NIHSS score, and GCS score. Bivariate correlation was analyzed via the Spearman coefficient. The asterisk indicates statistical significance (*p < 0.05, **p < 0.01).

It was revealed that the plasma MMP-2 content of ICH individuals was markedly reduced than healthy controls (38.02 ± 1.71 vs. 54.03 ± 2.15, *p* < 0.0001) (Figure 2A). To further understand the association of plasma MMP-2 content of ICH individuals and their clinical outcomes, their edema volume and NIHSS and GCS scores were analyzed. It was indicated that the plasma MMP-2 levels were increased in individuals with <10 mL (39.81 ± 2.23) of edema volume than in those with >20 mL (34.97 ± 3.13) and 10–20 mL (29.76 ± 2.93) of edema volume (*p* = 0.03) (Figure 2B). Furthermore, the plasma MMP-2 content was relatively reduced in individuals with higher NIHSS scores (*p* = 0.02) than those with lower scores. The plasma MMP-2 levels in individuals with <9 NIHSS scores (43.63 ± 3.28) were substantially increased than those with 9–20 scores (32.65 ± 2.79) and > 20 scores (36.12 ± 2.16) (Figure 2C). However, the plasma MMP-2 content of patients with >12 GCS scores (43.16 ± 2.84) was notably higher than that in those with <9 (33.67 ± 2.46) and 9–12 GCS scores (35.67 ± 2.98) (*p* = 0.03) (Figure 2D). The results of this investigation suggest that MMP-2 levels are significantly alleviated in the plasma of hemorrhagic patients and are closely related to edema volume. To further confirm the relationship of MMP-2 levels with edema volume and NIHSS and GCS scores, Spearman’s correlation coefficient analysis was conducted. Patients’ edema volume, NIHSS and GCS scores, and CT imaging diagnoses were utilized for analysis (Figure 3A). Subsequently, it was indicated that plasma MMP-2 levels were decreased markedly with increasing edema volume (*r* = −0.2187, *p* = 0.04) (Figure 3B). MMP-2 levels are negatively linked with NIHSS score (*r* = −0.2194, *p* = 0.03) (Figure 3C) and positively linked with GCS score (*r* = 0.2451, *p* = 0.01 Figure 3D).

### Plasma MMP-2 level and edema volume regression analysis

We have found correlation between MMP-2 level and edema volume. The degree of brain edema volume around the haematoma volume is associated with poor clinical outcome in patients with ICH. We used logistic regression analysis to investigate the prognostic value of MMP-2 levels in patients with ICH. First, the dependent variable was edema volume, MMP-2 level or variables (age, mRS, GCS, haematoma volume, NIHSS, plasma potassium level, hypertension, alcohol consumption, blood leucocyte count, current smoking) was independent variables, respectively. The univariate logistic regression analysis revealed that low MMP-2 level was associated with high edema volume (odds ratio [OR] = 0.33, 95% confidence interval (95%CI) [0.13 to 0.76], *p* = 0.01) (Table 2). Age, mRS, GCS, haematoma volume and NIHISS were also significantly associated with edema volume (all *p*-values <0.05, Table 2). Next step, we used multivariate analysis to further confirmed the prognostic value of plasma MMP-2 levels. The dependent variable was edema volume, independent variables for adjustment were age, mRS, GCS, haematoma volume, NIHSS, and plasma potassium level, these variables were simultaneously used in multivariate logistic analysis. The results revealed that reduced plasma MMP-2 level is associated with elevated edema volume (OR = 0.30, 95% CI [0.10 to 0.88], *p* = 0.03), age (OR = 4.36, 95% CI [1.48 to 14.45], *p* = 0.001), haematoma volume (OR = 6.96, 95% CI [2.34 to 23.22], *p* = 0.0008), plasma potassium level (OR = 0.32, 95% CI [0.10 to 0.92], *p* = 0.04) were independently associated with edema volume (Table 2). These results indicated that the plasma MMP-2 level may be a prognostic factor and decreased plasma MMP-2 level was associated with poor prognostic in patients with ICH.

**Table 2:** Univariate and multivariate logistic regression analysis of edema in patients with ICH.

Clinical characteristic	Univariate analysis		Multivariate analysis	
	*p*-value	OR(95% CI)	*p*-value	OR(95% CI)
Age, years	0.0507*	1.95 (0.01 to 1.67)	0.001**	4.36 (1.48 to 14.45)
MRS	0.02*	2.65 (1.11 to 6.32)	0.79	0.84 (0.22 to 3.01)
GCS	0.004**	0.20 (0.08 to 0.48)	0.27	0.4 (0.07 to 2.08)
Hypertension	0.14	1.97 (0.79 to 5.10)		
Haematoma volume	<0.0001**	5.98 (2.50 to 15.14)	0.0008**	6.96 (2.34 to 23.22)
Alcohol consumption	0.53	0.69 (0.21 to 2.18)		
MMP-2	0.01**	0.33 (0.13 to 0.76)	0.03*	0.30 (0.10 to 0.88)
Blood leucocyte count	0.17	1.76 (0.77 to 4.07)		
NIHISS	0.0004**	4.87 (2.06 to 12.05)	0.54	1.63 (0.33 to 8.36)
Current smoking	0.98	0.97 (0.11 to 8.43)		
Plasma potassium level	0.07	0.47 (0.20 to 1.08)	0.04*	0.32 (0.10 to 0.92)

mRS, Modified Rankin Scale; GCS, Glasgow Coma Scale; NIHSS, National Institutes of Health Stroke Scale; MMP-2, metalloproteinases-2.The asterisk indicates statistical significance (*p < 0.05, **p < 0.01).

### Relationship between plasma MMP-2 levels and mRS, blood leucocyte count, MMP-14, complement C4 levels of ICH patients

**Figure 4:**
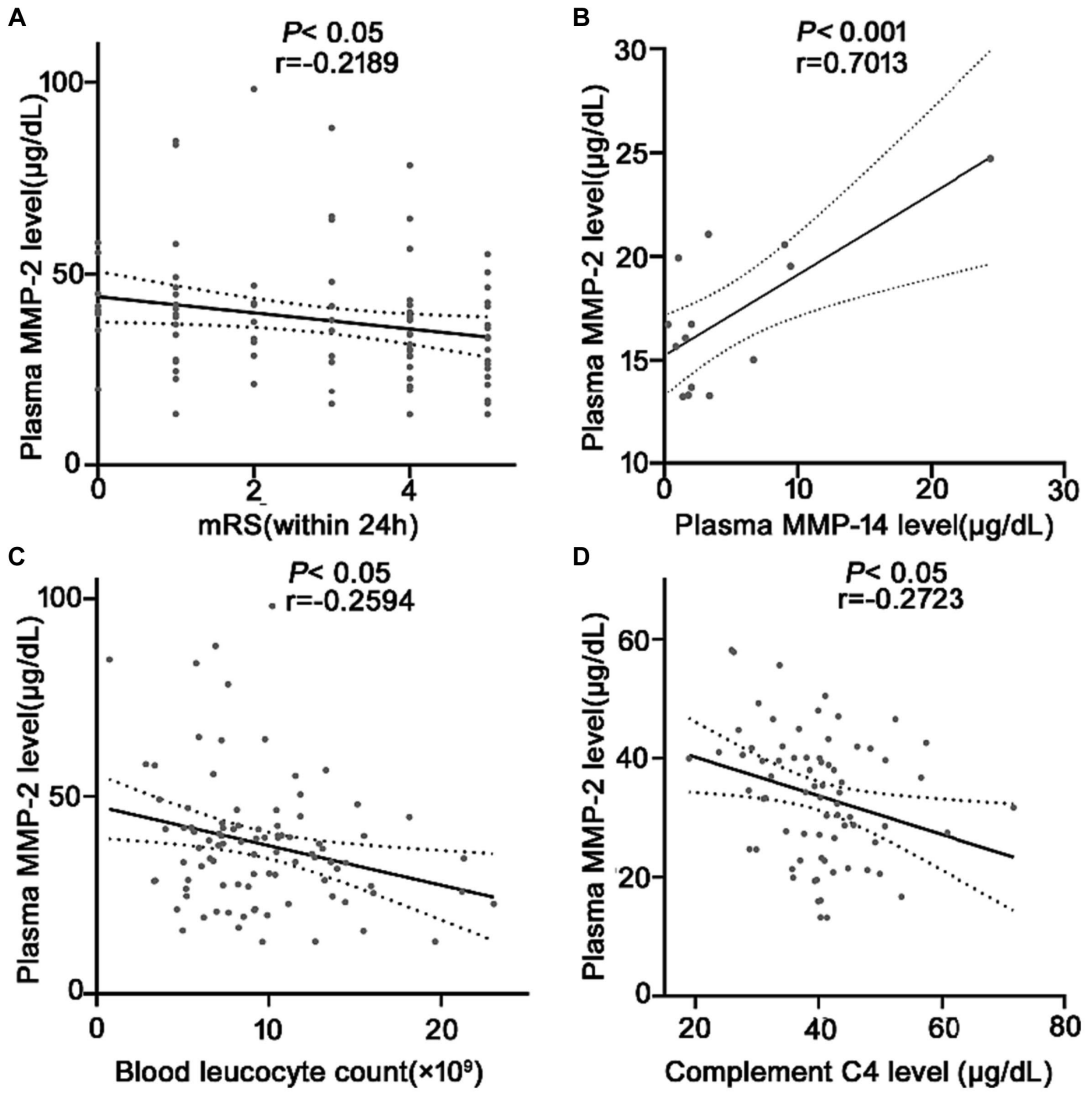
Correlation analysis between the plasma MMP-2 level and **(A)** mRS after ICH, **(B)** plasma MMP-14 levels (*n* = 14), **(C)** plasma blood cell count, and **(D)** plasma C4 levels at 24 h in patients with ICH. Bivariate correlation was analyzed via the Spearman coefficient. The asterisk indicates statistical significance (*p < 0.05, **p < 0.01).

A total of 45 (48.4%) patients had a poor prognosis (mRS scores 4–5) within 24 h after spontaneous ICH onset. mRS score assesses the neurological function recovery rate in ICH patients. The MMP-2 level is negatively correlated with the mRS score for an ICH within 24 h (*r* = −0.2189, *p* = 0.03) (Figure 4A). Suggesting a possible association between MMP-2 levels and prognosis just after ICH. Moreover, several links were identified among the researched molecules. Upon random detection of MMP-14 levels in 14 samples, it was revealed that MMP-2 and MMP-14 are correlated with each other (*r* = 0.7013, *p* = 0.005) (Figure 4B). Additionally, negative correlations were identified between MMP-2 and inflammatory parameters, blood leucocyte count (*r* = −0.2549, *p* = 0.01) (Figure 4C), and complement C4 level (*r* = −0.2723, *p* = 0.01) (Figure 4D).

## Discussion

This investigation identified that plasma MMP-2 levels in ICH individuals are markedly lower than in the healthy cohort, consistent with the finding of Jose et al., who indicated that the level of MMP-2 in ICH patients is decreased within 24 h (2). In addition, it was found that plasma MMP-2 levels are negatively correlated with NIHSS scores, and individuals with reduced plasma MMP-2 content have lower GCS scores, these suggest poor neurological function in ICH individuals (16). Furthermore, this investigation is the first to reveal that PHE volume is negatively correlated with plasma MMP-2 levels. One research reported that active MMP-2 level and edema volume were positively associated in 28 patients within 24 h after ICH (12), which is inconsistent with the data of this investigation. The possible difference might be because the samples and edema volume were collected from 93 ICH patients, which is more than 28 study size. In short, the data of this investigation confirmed that the plasma MMP-2 level of patients after ICH decreases and provide evidence for the association between MMP-2 level, neuropathic function, and early edema volume.

Furthermore, it was also revealed that the reduced plasma MMP-2 level was positively linked with the hemorrhagic severity and clinical outcomes. We firstly confirmed that plasma MMP-2 level was an independent prognostic factor assessed by logistic regression analysis. This data further confirms the role of reduced active MMP-2 levels on brain injury after ICH, but the specific mechanism needs further study. Massimiliano has reported that the MMP-2 might have protective activity against peri-hematomal neuroinflammation and tissue repair (12), but the mechanism is unknown. Many studies also reported that MMP-2 and MMP-9 are upregulated in rat ICH models, can cause SBI, and have a considerable clinical impact (6, 17). Here, it was revealed that MMP-2 level was negatively linked with mRS score in early-stage ICH patients. The mRS score was used to evaluate the patient’s neurological function at admission (18). The correlations of MMP-2 level and MMP-14 level was firstly observed in patients with ICH. MMPs can contribute to peri-hemorrhagic inflammatory response because MMPs open the BBB, and leukocyte migrates into the brain (7, 8). It was discovered that MMP-2 levels were negatively associated with blood leucocyte count and complement C4 level. Blood leucocyte count is an inflammatory marker (19). The complement system is associated with different immune reactions, such as cell lysis and the inflammatory response (20, 21). Per our knowledge, this is the first investigation that indicates the correlations of MMP-2 with blood leucocyte count and complement C4 level in ICH patients. Suggesting that MMP-2 might also be involved in the inflammatory response. However, further verification is needed. The function of Complement C4 is not limited to neuro-inflammation. The membrane-attack complex (MAC) comprises C5b-9 complement forms assembled after complement activation (22). Evidence shows that ICH is followed by the activation of complement cascade in the brain parenchyma (23). However, the insertion of MAC might also occur in glia, neurons, and endothelial cells, causing neuronal death and BBB leakage (24). Activation of MMP-2 involves a trimolecular complex composed on MMP-2, TIMP-2, and MMP-14 in membrane (25, 26). Formation of MAC cause the development of pores in the cell membrane (22). These pores may affect the formation of trimolecular complex of MMP-2, MMP-14 and tissue inhibitors to metalloproteinases-2 (TIMP-2) on the cell membrane, therefore affect the activation of MMP-2, and show a decrease in plasma MMP-2 levels. A report that C3a inhibited pro-MMP2 expression in cSCC cells by C3aR blockading has indeed been reported (27). Further research is needed to verify the formation of MAC blocking the contact between pro MMP-2, TIMP-2 and MMP-14 proteins on surface of cell and affecting the activation of MMP-2, and to identify the reasons for negatively correlation between complement C4 level and MMP-2 level. This article also has certain flaws, the data from patients with the potentially most striking edema development may be discarded in the study. In a word, this research is the first to indicate the relationship between plasma MMP-2 levels and complement C4 level, suggesting the possible involvement of complement in MMP-2 decrease and also providing a new direction to find the cause of the decrease in MMP-2 in patients with ICH.

## Conclusion

In conclusion, the plasma MMP-2 levels are substantially reduced in ICH patients. It was revealed that this decrease correlated with patients’ clinical results. We firstly confirmed plasma MMP-2 level may be a prognostic factor, and firstly investigated that plasma MMP-2 levels are positively linked with plasma MMP-14 and negatively linked with plasma C4 level and blood leucocyte count in ICH patients, which also suggests that MMP-2 might be involved in the inflammatory response. This investigation provides novel evidence for MMP-2 as a prognosis factor for ICH and for the relationship of MMP-2 with the inflammatory response in ICH patients.

## Data availability statement

The original contributions presented in the study are included in the article/supplementary material, further inquiries can be directed to the corresponding authors.

## Ethics statement

The studies involving humans were approved by the Institutional Review Boards at Affiliated Hospital of Jiujiang University (Grant No. IRB2022-JJU-032-21). The studies were conducted in accordance with the local legislation and institutional requirements. The participants provided their written informed consent to participate in this study. Written informed consent was obtained from the individual(s) for the publication of any potentially identifiable images or data included in this article.

## Author contributions

WY: Funding acquisition, Supervision, Writing – original draft, Writing – review & editing. JP: Conceptualization, Writing – review & editing. ZC: Writing – review & editing. HL: Writing – original draft. JY: Writing – original draft. YW: Writing – review & editing. MZ: Formal analysis, Writing – review & editing. MW: Conceptualization, Project administration, Supervision, Writing – original draft, Writing – review & editing.
